# Spiritual intelligence: a scoping review on the gateway to mental health

**DOI:** 10.1080/16549716.2024.2362310

**Published:** 2024-06-21

**Authors:** Cristina Teixeira Pinto, Lúcia Guedes, Sara Pinto, Rui Nunes

**Affiliations:** a Bioethics Department, Faculty of Medicine of the University of Porto, Porto, Portugal; b Centro Hospitalar de Entre Douro e Vouga, Intrahospital Palliative Care Team, Santa Maria da Feira, Portugal; c Nursing School of Porto, Porto, Portugal; d NursID, CINTESIS@RISE, Porto, Portugal

**Keywords:** Spiritual intelligence, spirituality, mental health, spiritual care, medical education, health education

## Abstract

Spiritual Intelligence (SI) is an independent concept from spirituality, a unifying and integrative intelligence that can be trained and developed, allowing people to make use of spirituality to enhance daily interaction and problem solving in a sort of spirituality into action. To comprehensively map and analyze current knowledge on SI and understand its impact on mental health and human interactions, we conducted a scoping review following the Joanna Briggs Institute methodology, searching for ‘spiritual intelligence’ across PubMedCentral, Scopus, WebOfScience, and PsycInfo. Quantitative studies using validated SI instruments and reproducible methodologies, published up to 1 January 2022, were included. Selected references were independently assessed by two reviewers, with any disagreements resolved by a third reviewer. Data were extracted using a data extraction tool previously developed and piloted. From this search, a total of 69 manuscripts from 67 studies were included. Most studies (*n* = 48) were conducted in educational (*n* = 29) and healthcare (*n* = 19) settings, with the Spiritual Intelligence Self Report Inventory (SISRI-24) emerging as the predominant instrument for assessing SI (*n* = 39). Analysis revealed several notable correlations with SI: resilience (*n* = 7), general, mental, and spiritual health (*n* = 6), emotional intelligence (*n* = 5), and favorable social behaviors and communication strategies (*n* = 5). Conversely, negative correlations were observed with burnout and stress (*n* = 5), as well as depression and anxiety (*n* = 5). These findings prompt a discussion regarding the integration of the SI concept into a revised definition of health by the World Health Organization and underscore the significance of SI training as a preventative health measure.

## Paper context

- **Main findings**: This scoping review of Spiritual Intelligence found positive correlations with resilience, general, mental and spiritual health, emotional intelligence, and favourable social behaviours and communication strategies, and negative correlations with burnout, stress, depression, and anxiety.
- **Added knowledge**: Spiritual Intelligence is an all-inclusive way to approach spirituality from a practical, daily problem-solving perspective that can be trained with several benefits for personal overall health, while also fostering substantial personal growth in social behaviors and skills.
- **Global health impact for policy and action**: Spiritual Intelligence training is urgently needed and should be integrated into global educational programs from early childhood as a health promotion strategy aiming to foster a more resilient and compassionate society.

## Background

Since the first steps of the multiple intelligences theory by Gardner [1], the discussion on this matter has ever since been around the concept of independent, yet intertwined, mental skill sets that work in different dimensions of human existence. Initially, Gardner’s theory set eight main intelligence domains: visual-spatial; verbal-linguistic; musical-rhythmic; logical-mathematical; interpersonal; intrapersonal; naturalistic and bodily kinesthetic. This fruitful work later opened new horizons to the discussion of a new concept, entitled ‘Spiritual Intelligence’ (SI), in the late 90s, as the discussion on an intelligence domain concerning existential matters grew.

Acknowledging spirituality as the self-concept of one’s soul, a search for the sacred or transcendent and the personal vertical relationship one establishes with what one deems sacred, it is a separate concept from SI (though related). Defined as the personal skill to bring one’s own spirituality and the transcendental relationship with the sacred into daily problem solving, SI enhances one’s mental fortitude by expanding personal metaviews on daily interactions, broadening senses of purpose and meaning, and drawing spiritual capital from social participation, pushing the person forward towards their goals [2]. Furthermore, SI is not to imply any religious belief or practice one chooses to integrate in their spiritual existence.

Closely linked to emotional intelligence (EI), SI facilitates the cognitive shift from the empathic ability to read and understand emotions to the compassionate ability to take action towards the ease of suffering without becoming overwhelmed by it [3]. Almost as if EI reads, the emotions but SI further ‘reads the room’ or the ‘full picture’, thus the metaview.

The core SI dimensions [2] are: (1) critical existential thinking, as the ability to answer deep existential questions in an open-minded and bias-free personal way, free from judgement and preconceptions allowing for self-evolution and self-actualization; (2) personal meaning production, as the ability to draw meaning from all good or bad, complex or simple, material or immaterial daily interactions and by making them meaningful integrating them in a positive and healthy way in one’s personal history and elevating one’s sense of well-being, meaning and purpose in life [4,5]; (3) transcendental awareness, as the ability to understand the world from a metaview perspective, aware both of the individual’s participation in the universe and the universe within the individual, making every decision and life event more than a self-directed event and putting it to a larger scale that makes one unique and relevant to the universe in one choices, thus responsible for them, but also a reflect of the universal interactions, thus not at fault for what overruns us; and (4) conscious state expansion, as the ability to enter, at one’s own will, into deeper states of spiritual attention and concentration (as in meditation) helping to capture from specific moments more than meets the eye and enabling a deeper connectedness to the sacred, nature or even other people, raising one’s fulfilment and sense of belonging.

Even though spirituality is an inherent part of the human being, the willingness to tap into it in a self-aware and self-constructive way is what makes SI. This ability to voluntarily explore the spiritual self and make it grow in individual interactions makes for a more fulfilling and resilient existence [6].

Reinforcing the SI original concept, many quantitative studies throughout the past two decades have shown significant protective impact of SI concerning environmental, interpersonal and work-related stressors [7–12].

At this time, and considering a preliminary search on PubMedCentral (PMC), no scoping reviews (published or ongoing) were found on this topic. Therefore, a Scoping Review (ScR) was conducted using the methodology proposed by the Joanna Briggs Institute (JBI) for Scoping Reviews [13]. The aim of this review was to map and analyze the current knowledge in this field by synthesizing existing quantitative studies, with the goal of establishing the known ramifications of SI and better understanding its impact on mental health and human interactions.

## Review questions

This ScR aims to answer the general problematic:

What is known from the literature about the influence of Spiritual Intelligence on human behavior and mental health?

More specifically, our review questions are:

1. In what settings and populations is SI being studied?
2. Which instruments for SI measurement are being used?
3. What sociodemographic factors relate to SI?
4. What positive correlations to SI have been reported?
5. What negative correlations to SI have been reported?

## Eligibility criteria

### Participants

This ScR included any studies, with no age or sociodemographic background restrictions for the included samples or settings.

### Concept

The included studies concern or directly address SI, as defined by King [2]. Studies merely focusing on the general concept of spirituality were excluded.

### Context

We considered studies on a vast spectrum of settings and people, undertaken in multiple areas of knowledge, including but not limited to healthcare and psychology.

### Types of studies

This ScR considered for inclusion any quantitative studies that use properly validated SI evaluation instruments and reproducible methodology (aims, inclusion criteria, sampling and instrument validation and reliability).

Text, proceedings, conference or opinion papers, abstracts, reviews, mixed methods and qualitative studies were excluded. Studies published in predatory journals were considered for inclusion as long as they complied with the criteria and quality assessment.

## Methods

This ScR was conducted in accordance with the Joanna Briggs Institute (JBI) methodology [13] and reported according to the PRISMA extension for scoping reviews (PRISMA-ScR) [14].

### Search strategy

Studies published in English, French, Spanish or Portuguese were included, since these are languages the authors are fluent in.

Studies published up until 1 January 2022, were included to document the evolutionary timeline of the SI concept. A search for ‘Spiritual Intelligence’ (search term) was conducted on PMC, Scopus, WebOfScience and PsycInfo databases and the search strategy was adapted for each included database.

### Study/source of evidence selection

**Figure 1.**
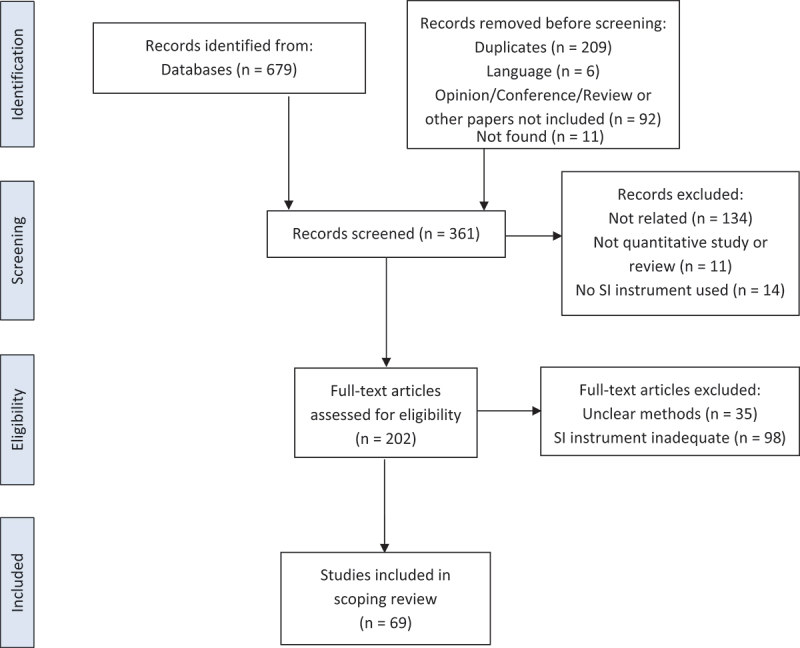
PRISMA-ScR flow diagram [14].

Following the search, all identified citations were uploaded to Mendeley® and duplicates removed. Titles and abstracts were screened by two independent reviewers (CTP, LG) for assessment against the inclusion criteria. The full text of selected citations was then assessed in detail by two reviewers (CTP, LG). Any disagreements between the reviewers at each stage of the selection process were discussed among the team or reviewed by a third reviewer (SP, RN). The results of the search and study inclusion process were reported according to the PRISMA-ScR flow diagram (Figure 1) [14].

### Data extraction

Data were extracted from the included papers by two independent reviewers (CTP, LG) using a data extraction tool developed by the reviewers. Prior to the study, the data extraction tool was pilot tested and trained by the reviewers with a total of 10 manuscripts with further cross-checking of the extracted data for consistency. The extracted data included various study details (authors, year, journal, country), as well as information on participants, study settings, aims, methodologies, SI instruments employed (including reliability and validation data), and key findings pertinent to the review objectives (including both positive and negative correlations to SI).

## Results

Searches of electronic databases identified 470 hits, excluding duplicates (Figure 1). After screening, 159 hits were excluded, which identified 202 eligible manuscripts. This resulted in 69 included manuscripts reporting on 67 studies.

**Figure 2.**
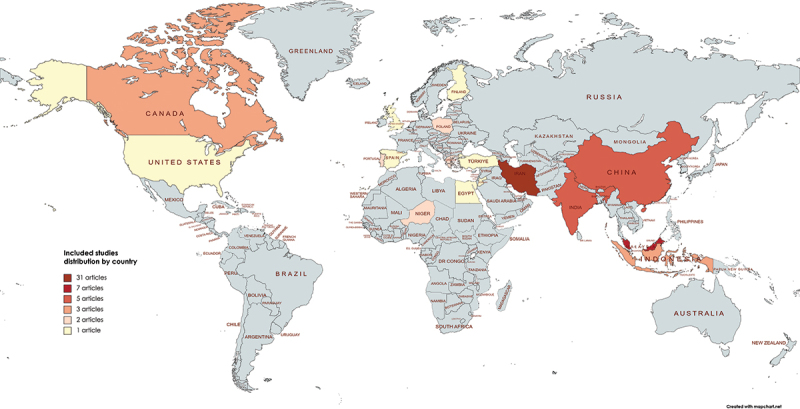
Geographic distribution of included studies.

Among the 67 studies included for review, 32 were conducted in the Middle East (*n* = 34 manuscripts), 20 in Asia, nine in Europe, four in North America and two in Sub-Saharan Africa (Figure 2).

**Figure 3.**
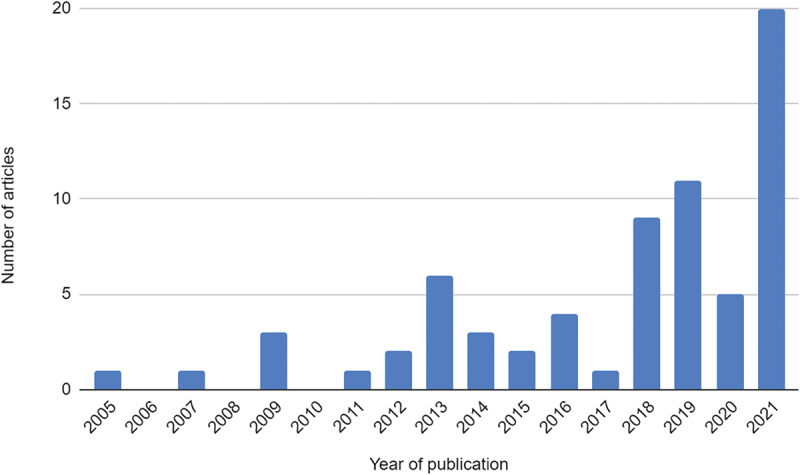
Number of included studies by year of publication.

Until 2011, only five publications complied with our above-mentioned study quality assessment criteria; from 2011 until 2016, 14 studies; from 2016 to 2021, 30 studies and in 2021 alone a total of 20 relevant publications were found (Figure 3).

Concerning the study design, most were correlational (*n* = 33) or descriptive (*n* = 18), with nine reporting on tool validations/psychometric assessment and only two quasi-experimental.

### Settings and populations

Regarding the setting in which the studies took place, the educational system was the most frequent (*n* = 29), with the most frequently studied population being university students (*n* = 9456) and teachers (*n* = 1880). The second most frequent setting was the healthcare system (*n* = 19). Participants included 2948 nurses, 2100 patients and 1069 healthcare students. Among the patients, most were diagnosed with diabetes (*n* = 836), coronary diseases (*n* = 398) or were pregnant women (*n* = 379).

Studies involving the general population (*n* = 10) included 6577 individuals while those focusing on organizations (*n* = 5) included a total of 5150 various employees (Table 1).

**Table 1.** Summary of main findings in the included studies.

Author (year)	Type of study	Setting	Sample/Participants	Aims	SI instrument	Main results
Tirri et al. (2005)	Quantitative (cross-sectional descriptive)	Military	195 peacekeepers	SI and multiple intelligences profile	Own instrument	Scale validation
Yang et al. (2007)	Quantitative (cross-sectional descriptive-inferential)	Healthcare	130 nurses	SI profile among nurses and relationships with individual characteristics and religion	PsychoMatrix Spirituality Inventory, Wolman (2001)	Nurses with religious affiliation have higher SI scores and religious beliefs correlate to SI factors
King et al. (2009)	Quantitative (scale validation)	Education	619 psychology undergraduate students	Developing and validating a SI instrument	Own instrument (SISRI)	Scale validation; SI was positively correlated with meaning of life, metapersonal, mysticism, intrinsic-extrinsic religiosity and desirable responding
Martin et al. (2009)	Quantitative (cross-sectional descriptive)	Education	532 university business students	SI and EI effect on work-performance	Adapted from SI, Ashmos and Duchon (2000)	EI and SI are statistically correlated
Yang et al. (2009)	Quantitative (cross-sectional descriptive-inferential)	Healthcare	524 nurses	SI profile and demographics in two different Chinese societies	PsychoMatrix Spirituality Inventory, Wolman (2001)	Nurses with vocational schooling and religious affiliation had higher SI; age, childhood spirituality and social system were significant predictorsof SI
Narayanan et al. (2011)	Quantitative (cross sectional descriptive)	Education	220 Christian adolescents	SI and resilience among Christian youth	Integrated SI Scale, Amram and Dryer (2008)	Of the 22 SI capabilities studied, 16 were significantly positively correlated with resilience and explain 57% of its variance
King et al. (2012)	Quantitative (cross-sectional descriptive-correlational)	General Population	420 (362 university students & 58 general population)	Relationship between EI and SI, empathy as a potential component of SI	SISRI-24, King and DeCicco (2009)	Assessing Emotions Scale (Schutte et al., 1998) and Trait Meta Mood Scale (Salovey et al., 1995) displayed significant low-to-moderate positive correlations with the SISRI-24; Multidimensional Emotional Empathy Scale (Caruso & Mayer, 1998) showed no relation to SISRI-24
Honarmand et al. (2012)	Quantitative (cross-sectional descriptive-correlational)	Education	221 adolescents	SI and happiness of adolescents in Iran	Spiritual sensitivity scale	Positive relation between happiness and SI; SI can predict 67% of variations in happiness scores
Kaur et al.. (2013)	Quantitative (cross-sectional descriptive)	Healthcare	448 nurses (& 348 patients)	Nurses’ caring behavior and SI, EI, psychological ownership and burnout	SISRI-24, King and DeCicco (2009)	Positive relationship between SI and EI; nurses with higher SI have higher levels of psychological ownership towards their jobs which also mediates the positive relationship between SI and caring behavior;
Azizollah et al. (2013)	Quantitative (cross-sectional descriptive-correlational)	Education	250 university students	SI and EI effect on student achievement	SISRI-24, King and DeCicco (2009)	Interrelationships between different types of intelligence (SI, EI) and achievement
Shahbakhsh et al. (2013)	Quantitative (cross-sectional descriptive-correlational)	Healthcare	100 patients referred to the addiction center	SI and resilience effect on withdrawal time of individuals treated with methadone	SISRI-24, King and DeCicco (2009)	Positive correlation between resilience and SI and withdrawal time and SI
Rani et al. (2013)	Quantitative (cross-sectional descriptive-inferential)	Healthcare	506 nurses	SI effect on nurses’ work performance	Integrated SI Scale, Amram and Dryer (2008)	Positive correlation between SI and work performance; nurse’s tenure is moderating factor between SI and work performance
Hassan et al. (2013)	Quantitative (cross-sectional descriptive-correlational)	Education	247 adolescents	SI, EI and mental health in adolescents	Integrated SI Scale, Amram and Dryer (2008)	SI highly positively related with EI; SI has significant indirect effect via EI on reducing mental health problems explaining 68% of variance; SI and its dimensions were highly negatively related with mental health problems
Azizi et al. (2013)	Quantitative (cross-sectional descriptive-correlational)	Education	120 EFL students	SI and EFL learners’ use of vocabulary learning strategies	SISRI-24, King and DeCicco (2009)	Significant correlation between SI and metacognitive strategies and social strategies; females showed higher spiritual intelligence scores
Khosravi et al. (2014)	Quantitative (cross-sectional descriptive-correlational)	Education	307 university students	SI, resilience and perceived stress among students	SISRI-24, King and DeCicco (2009)	Positive and significant relationship between SI and resilience; SI predicts 10% of resilience variance; negative and significant relationship between SI and perceived stress; SI predicts 11% of perceived stress variance
Khayyam Nekouei et al. (2014)	Quantitative (cross-sectional descriptive-correlational)	Healthcare	398 patients with coronary heart disease	Psychological risk and protective factors and quality of life in patients with coronary heart disease	SQ-21 Wigglesworth (2021)	Among the protective factors of coronary heart disease, SI with a factor loading of 0.58 had the maximum value
Saeedi et al. (2014)	Quantitative (cross-sectional descriptive-correlational)	Education	115 female students	SI and religious orientation of students	Own instrument	Positive correlation between SI and religious orientation; last year students have higher SI
Anwar et al. (2015)	Quantitative (cross-sectional descriptive-correlational)	Organizations	107 employees of manufacturing and service industries	SI effect on employee citizenship behavior in manufacturing and service employees	Adapted from SISRI-24 King and DeCicco (2009)	Organizational citizenship behavior is positively correlated with SI, which answers for 24.8% of its variation
Mahasneh et al. (2015)	Quantitative (cross-sectional descriptive)	Education	682 university students	SI profile and link to personality traits	SISRI-24, King and DeCicco (2009)	Positive significant relationship between SI and personality traits which explains 12% variance in critical existential thinking, 10,7% in personal meaning production, 5,7% in transcendental awareness and 9,3% in conscious state expansion SI dimensions
Chan et al. (2016)	Quantitative (scale validation)	Education	213 university students	Developing and validating a SI instrument	SISRI-24, King and DeCicco (2009)	Scale validation; SI was positively correlated with meaning in life, metapersonal self-construal and satisfaction with life
Jorge et al. (2016)	Quantitative (scale validation)	General population	718 adults	Developing and validating a SI instrument	Integrated SI Scale, Amram and Dryer (2008)	Scale validation
Amirian et al. (2016)	Quantitative (cross sectional descriptive-correlational)	Education	384 university students	SI, general health and happiness among students	SISRI-24, King and DeCicco (2009)	Higher SI positively relates and predicts better general health and happiness
Geram, Kazem (2016)	Quantitative (cross-sectional descriptive-correlational)	Education	300 male teachers	SI and EI effect on social adjustment of male high-school teachers	SI Questionnaire, Badii et al. (2000)	Significant positive relation between social adjustment and SI
Udin et al.. (2017)	quantitative (cross sectional descriptive-correlational)	Organizations	239 automobile engineers	SI and intrinsic motivation effect on affective commitment and engineer performance	Own instrument	SI positively affects affective commitment and engineer performance
Fazlolah, MirDrikvand (2018)	Quantitative (cross-sectional descriptive-correlational)	Education	290 high school teachers	identity styles and SI of high school teachers	SI Questionnaire, Abdullah Zadeh (2008)	Positive correlation between SI and informative identity style, normative style and commitment identity
Riahi et al. (2018)	Quantitative (quasi-experimental)	Healthcare	82 ICU nurses (40 experimental vs 42 control group)	SI training and nurses’ competence in spiritual care	SISRI-24, King and DeCicco (2009)	Higher SI after intervention; higher spiritual care competence after intervention; mean scores of spiritual care competence in the experimental group were higher than the control group;
Malini et al. (2018)	Quantitative (cross-sectional descriptive)	Education	523 undergraduate students	students SI and motivation and the mediating effects of gender	SI, Hildebrant (2011)	Moderate influence of motivation on SI; all SI domains are significantly correlated to the overall motivation
Abdollahpour et al. (2018)	Quantitative (cross-sectional descriptive)	Healthcare	245 pregnant women	SI and happiness effect on fear of childbirth in pregnant women	SISRI-24, King and DeCicco (2009)	People with higher score of SI are happier; negative correlation between SI with fear of childbirth; mothers who had no fear of childbirth had a higher level of SI
Antunes et al. (2018)	Quantitative (scale validation)	General Population	767 adults	Developing and validating an SI instrument	SISRI-24, King and DeCicco (2009)	Scale validation
Zarrinabadi et al. (2018)	Quantitative (cross-sectional descriptive-correlational)	Education	260 university medical librarianship students	Validating an SI instrument	SISRI-24, King and DeCicco (2009)	Internal validity of SI instrument confirmed
Baloochi et al. (2018)	Quantitative (cross-sectional descriptive-correlational)	Education	300 bachelor students of medical sciences	SI and aggression among Iranian medical sciences students	SISRI-24, King and DeCicco (2009)	Significant positive correlation between SI and older age, married status and management study field; negative relationship between SI and aggression, predicting 29,5% of variation
Rahmanian et al. (2018 & 2019)	Quantitative (cross-sectional descriptive-correlational)	Healthcare	200 adolescents with type 1 diabetes	SI effect on selfmanagement in adolescents with type 1 diabetes	SISRI-24, King and DeCicco (2009)	SI can predict 4.7% of diabetes selfmanagement changes and its effect on diabetes selfmanagement with special impact of SI dimension of personal meaning production
				SI and selfefficacy in diabetic adolescents		significant relationship between SI and selfefficacy
				SI and demographics in diabetic adolescents		none of the variables were related
Sharma, Sonia (2019)	Quantitative (graphic survey method)	General population	530 women (280 professional vs 250 non-professional workers)	SI, EI and life satisfaction among professional and non-professional working women	SI Scale, Dhar and Dhar (2010)	professional working women with high spiritual intelligence are better adjusted than their counterparts
Hatami et al. (2019)	Quantitative (cross-sectional descriptive)	Healthcare	134 pregnant women	SI and resilience relationship with fear of childbirth in pregnant women	SISRI-24, King and DeCicco (2009)	significant correlation between SI and resilience; SI higher in natural childbirth than cesarean; SI higher in nulliparous women than multipara; SI higher with education level; age increases level of resilience and SI
Polemikou et al. (2019)	Quantitative (cross-sectional descriptive)	Organizations	182 first responders	SI as a moderator between death anxiety and dissociative post-traumatic stress disorder among first responders	SISRI-24, King and DeCicco (2009)	weak positive associations between SI and death anxiety as well as with dissociative experiences; SI and death anxiety predict/explain 77% of the variance in dissociative experiences; death anxiety was significantly related to dissociation, and SI significantly moderated that relationship with higher SI in high-level dissociation group
Polemikou et al. (2019)	Quantitative (scale validation)	General population	1777 adults	Developing and validating an SI instrument	SISRI-24, King and DeCicco (2009)	scale validation; SI was positively correlated with meaning in life and resilience; inverse correlation between SI and nonreligious-nonspiritual scale (higher scores represent weak institutional religiousness/individual spirituality)
Dami et al. (2019)	Quantitative (quasi-experimental)	Education	64 students with final exams (32 intervention vs 32 control groups)	Effectiveness of depression, anxiety, stress and SI focused group counseling for students facing final exams	SISRI-24, King and DeCicco (2009)	SI increased after the intervention compared to the control group, mainly due to the critical existential thinking and personal meaning production components; after the intervention also depression, anxiety and stress experienced a significant decrease compared to the control group
Mosavinezhad et al. (2019)	Quantitative (cross-sectional descriptive)	Education	300 university psychology students	SI and personal beliefs effect on social anxiety among students	SISRI-24, King and DeCicco (2009)	negative correlation between SI and social anxiety; existential critical thinking, personal meaning and purpose, and transcendental consciousness dimensions of SI inversely predict social anxiety in up to 42% of its variance
Feng et al. (2019)	Quantitative (scale validation)	General population	605 university students and employees	Developing and validating an SI instrument	own instrument (Scale of SI for the Chinese Context)	scale validation
Ghalaychi et al. (2019)	Quantitative (cross-sectional descriptive-correlational)	Healthcare	200 midwives (& their patients with vaginal births)	Spiritual health and SI of midwifery students and satisfaction of patients with vaginal childbirth	SISRI-24, King and DeCicco (2009)	positive correlation between SI of midwives and mothers’ satisfaction with vaginal childbirth
Ebrahimi Barmi et al. (2019)	Quantitative (cross-sectional descriptive-correlational)	Healthcare	163 rehabilitation experts	SI and resilience of rehabilitation staff	SISRI-24, King and DeCicco (2009)	positive relationship between SI and resilience in rehabilitation staff
Pahlevan Sharif et al. (2019)	Quantitative (cross-sectional descriptive-correlational)	Military	211 male Iran–Iraq war veterans with war-related disabilities	SI, spiritual well-being and death anxiety among Iranian veterans	SISRI-24, King and DeCicco (2009)	significant positive relationship between SI and death anxiety
Sondakh et al. (2020)	Quantitative (scale validation)	Education	548 undergraduate students	Developing and validating a SI instrument	own instrument (Hi-ACT)	scale validation
Arnout et al. (2020)	Quantitative (cross-sectional descriptive)	General population	761 unemployed persons	Relationship between unemployment stress, SI and mental health components	SI scale (SIS-27), Arnout (2016)	factors of spiritual intelligence (spiritual mindfulness, spiritual abilities, and spiritual presence) intermediate between unemployment stress and mental health of unemployed adults
Anwar et al. (2020)	Quantitative (cross-sectional descriptive)	Education	250 Muslim business administration university students	Developing and validating a SI instrument and SI relationship to EI among students	own instrument (SI from an Islamic perspective)	significant relationship between SI from an Islamic perspective and EI
MARTÍN-SÁNCHEZ et al. (2020)	Quantitative (scale validation)	General population	528 adults	Developing and validating a SI instrument	Cuestionario de Inteligência Espiritual	gender differences in SI domains profiles (women higher: transcendence, compassion, forgiveness and gratitude; men higher: freedom and pain); conscience, transcendence, freedom and pain SI domains increase with education; different religious beliefs promote different SI profiles; shared living relates to higher transcendence, meaning, compassion and pain SI domains; significant positive correlation to resilience
Arsang-Jang et al. (2020)	Quantitative (cross-sectional descriptive-correlational)	Healthcare	376 nurses	SI and ethical decision making in hospital nurses	SISRI-24, King and DeCicco (2009)	postconventional nursing principled thinking relates positively to SI dimensions of critical existential thinking and personal meaning production, as well as total SI while preconventional nursing principled thinking relates negatively to the same variables
Behzad et al. (2021)	Qquantitative (cross-sectional descriptive)	Healthcare	145 university healthcare students (children of war martyrs or veterans)	Clinical competence and SI in students who are children of war victims	SI Questionnaire, Kazdin et al. (1986)	highest mean score of SI in nursing students and lowest in radiology students; direct, positive, and significant linear relationship between SI and clinical competency
Badrudin et al. (2021)	Quantitative (cross-sectional descriptive-correlational)	Healthcare	924 nursing and medical students	SI and spiritual health amongst Muslim students during the COVID-19 pandemic	SISRI-24, King and DeCicco (2009)	positive relationship between SI and spiritual health
Liu et al. (2021)	Quantitative (cross-sectional descriptive-correlational)	Education	569 primary school teachers	Mediating effect of SI between awe and life satisfaction among primary school teachers and ethnicity impact	Integrated SI Scale, Amram and Dryer (2008)	SI differs among teachers with different professional titles (third-grade title were higher than the others); working location significantly affects spiritual intelligence (highest SI for county); age and awe positively correlated with SI; SI plays a completely mediating role between awe and life satisfaction
Rahmawaty et al. (2021)	Quantitative (cross-sectional descriptive-correlational)	Organizations	196 microfinance employees	SI and EI effect on employee performance and the mediating role of communication competence	Zohar and Marshall (2000)	positive and significant influence of SI on communication competence and employee performance
Abdolrezapour et al. (2021)	Quantitative (explanatory sequential mixed-methods)	Education	47 female EFL students (22 intervention vs 25 control group)	SI intervention impact on EFL learners’ willingness to communicate and SI	SISRI-24, King and DeCicco (2009)	learners improved their willingness to communicate and their SI over time as a result of the intervention, with better performance of the experimental group on the posttest
Gera et al. (2021)	Quantitative (cross-sectional descriptive) and exploratory qualitative	Education	125 MBA students	SI impact on students’ academic performance	SISRI-24, King and DeCicco (2009)	SI domains of critical existential thinking and personal meaning production have a positive effect on academic performance explaining 19,9% of its variance
ÖZSARI et al. (2021)	Quantitative (cross-sectional descriptive)	Sports	31 physically handicapped badminton players	SI profile of physically disabled badminton players	SISRI-24, King and DeCicco (2009)	personal meaning generation dimension of SI was higher in male badminton players; personal meaning generation, awareness and conscious state expansion dimensions of SI were higher with age and married status; players with 3–5 years of sports experience had significantly lower SI scores
Paweł et al. (2021)	Quantitative (scale validation)	General population	833 adults	Validity of a general factor of SI as a psychological construct	SISRI-24, King and DeCicco (2009)	model did not fit to the data
Singla et al. (2021)	Quantitative (cross-sectional descriptive-correlational)	Education	451 college teachers	SI and quality of work life of college teachers and the mediating role of psychological capital	SISRI-24, King and DeCicco (2009)	relationship between SI and quality of work life is highly significant; psychological capital is a partial mediator in this relationship
Ahmadi et al. (2021)	Quantitative (cross-sectional descriptive-correlational)	Education	510 undergraduate nursing students	SI and spiritual care in nursing students	SISRI-24, King and DeCicco (2009)	positive correlation between competence in spiritual care and SI, in all dimensions of both concepts; SI higher with age, married status, clinical practice experience and higher academic year; SI explains 7% of the variance in competence in spiritual care
Hojat et al. (2021)	Quantitative (cross-sectional descriptive-correlational)	Healthcare	344 nurses	SI and professional self-concept among Iranian nurses	SI Questionnaire, Abdullah Zadeh (2008)	positive correlation between professional self-concept and SI, except for leadership subcategory; SI explains 13.3% of the changes in professional self-concept
Bhandari et al. (2021)	Quantitative (cross-sectional descriptive)	Yoga institute	70 Hindu ascetics	SI and distress of Hindu ascetics following material/sensual restrains and selfless service	SISRI-24, King and DeCicco (2009)	SI could account for a 14.4% variance in physical distress, 9.9% variance in psychological distress and 11.3% variance in total distress as a predictor; psychological distress can be positively predicted by conscious states expansion dimension of SI; high negative correlation between SI (mainly transcendental awareness dimension) and physical distress
Oyewunmi et al. (2021)	Qquantitative (cross-sectional descriptive-correlational)	Education	216 academic and administrative staff	SI effect on job performance, commitment and satisfaction	SISRI-24, King and DeCicco (2009)	Positive relationships between SI and job performance, job commitment, and job satisfaction
Ajele et al. (2021)	Quantitative (cross-sectional descriptive)	Healthcare	636 adult diabetics	SI and mindfulness effects and mediating role between emotional dysregulation, depression and mental well-being in persons with diabetes	SISRI-24, King and DeCicco (2009)	Mental well-being, depression, emotional dysregulation and mindfulness positive significantly correlated with SI; significant and positive direct effects of depression on mental well-being which was significantly moderated by mindfulness and SI; significant indirect relationship between depression and mental well-being via emotional dysregulation, which was significant and positively moderated by SI; significant positive direct relationship between SI and mindfulness explaining 8% of its variance; significant positive direct relationship between SI and mental well-being
Dargahi et al. (2021)	Quantitative (cross-sectional descriptive)	Education	267 school workers (181 employees & 86 management)	Ideal hybrid intelligence and employees’ organizational commitment	SISRI-24, King and DeCicco (2009)	Significant relationship between cultural, spiritual and moral intelligence, which constitute the dimensions of ideal hybrid intelligence; significant correlation of this ideal hybrid intelligence and employees’ organizational commitment; SI significantly affects employees’ organizational commitment
Mróz et al. (2021)	Quantitative (cross-sectional descriptive-correlational)	General population	605 adults	SI and forgiveness	SISRI-24, King and DeCicco (2009)	All SI dimensions positively related to forgivingness dimensions; different aspects of dispositional forgiveness mediated the link between two SI dimensions (personal meaning production and transcendental awareness) and episodic forgiveness, measured as a revenge and benevolence motivation, these two SI dimensions also related to a greater disposition to forgive with negative correlation to revenge and positive correlation to benevolence towards a specific offender; SI dimension of critical existential thinking negatively correlated with reduction of unforgivingness of situations; personal meaning production ad transcendental awareness dimensions of SI showed inverse correlation with revenge motivation
Alamanda et al. (2021)	Quantitative (cross-sectional descriptive-correlational)	Organizations	4125 manufacturing and serving employees	SI and organizational citizenship behaviors	PsychoMatrix Spirituality Inventory, Wolman (2001)	Positive relationship between SI and organizational citizenship behavior
Aliabadi et al. (2021)	Quantitative (cross-sectional descriptive-correlational)	Healthcare	338 nurses	SI and nurses’ empathy with COVID-19 patients	SISRI-24, King and DeCicco (2009)	Positive relationship between empathy and SI; SI explains 39,6% of empathy variation
Parattukudi et al. (2021)	Quantitative (cross-sectional descriptive-correlational)	Healthcare	39 mental health clinic patients	SI and depression	SISRI-24, King and DeCicco (2009)	Negative relationship between SI and depression, with significant gender differences in SI profile correlations
Pishghadam et al. (2021)	Quantitative (cross-sectional descriptive-correlational)	Education	270 EFL teachers	Rractance and SI effect on EFL teachers burnout	SISRI-24, King and DeCicco (2009)	SI negatively correlates to psychological reactance and burnout; SI partially mediates the relationship between reactance and burnout

### SI measuring instruments

The Spiritual Intelligence Self Report Inventory (SISRI-24) [2] was the most used SI measuring instrument (*n* = 39), with validation data from 11,331 individuals representing a wide variety of socio-demographic backgrounds, and demonstrating high reliability (all 39 studies reported Cronbach’s α > 0,60; of which 76,9% with α > 0,80) (Table 2). The Integrated Spiritual Intelligence Scale (ISIS) [15] was used in five studies representing a total validation population of 2142, with not so strong reliability data (reported Cronbach’s α ranging from 0,50 to 0,97).

**Table 2.** SISRI-24 worldwide validation data from selected studies.

Geographical area	Validation population	α Cronbach
North America Canada	Total population 1039 619 undergraduates 420 adults and university students	α 0,92 to 0,94 (total) α 0,92 (total) α 0,94 (total)
Europe Greece Portugal Poland England	Total population 4699 1794 general population 767 general population (80% university students) 833 general population 605 general population 700 college teachers	α 0,92 (total) α 0,92 (total) α 0,84 to 0,86 (dimensions) α 0,77 to 0,88 (dimensions) α 0,77 to 0,88 (dimensions) α 0,74 to 0,87 (dimensions)
Middle East Turkey Jordan Iran	Total population 3855 57 handicapped athletes 716 university students 250 university students 384 university students 10 adolescents 70 addiction patients 260 university students 200 midwives 300 medical students 25 rehabilitation experts 120 university students 211 male war veterans 300 university students 47 female English as Foreign Language students 270 English as Foreign Language teachers 630 undergraduate nursing students 5 cultural and theology experts	α 0,68 to 0,92 (total) α 0,88 (total) α 0,86 (total) α 0,87 (total) α 0,89 (total) α 0,90 (total) α 0,67 (total) α 0,92 (total) α 0,92 (total) α 0,75 (total) α 0,92 (total) α 0,86 (total) α 0,68 (total) α 0,89 (total) α 0,86 (total) α 0,87 to 0,91 (dimensions) α 0,91 (total) α 0,87 (total)
Africa Nigeria	Total population 500 500 academic and administrative staff	α 0,91 (total) α 0,91 (total)
Asia Hong Kong India	Total population 408 213 undergraduate students 125 MBA students 70 Hindu Ascetics	α 0,87 (total) α 0,87 (total) α 0,87 (total) α 0,89 to 0,95 (dimensions)
SouthEast Asia Indonesia Malaysia	Total population 830 240 Christian university students 448 nurses 112 employees 30 nursing and medical students	α 0,88 to 0,92 (total) α 0,92 (total) α 0,92 (total) α 0,88 α 0,78 to 0,84 (dimensions)

### Sociodemographics and SI

Reviewing the main findings from the included studies, the most frequently identified demographics related to SI were higher age (*n* = 7), religious affiliation (*n* = 6), and married status (*n* = 4). Other identified sociodemographic factors that favor SI included gender (with females reporting higher SI), higher education, childhood spirituality, a social system with fewer political constraints, ethnic attributes, geographic location (county living reported higher SI than country or towns) and community or shared living.

### Positive correlations to SI

The most frequently identified attributes significantly positively correlated to SI were: resilience (*n* = 7); general, mental and spiritual health (*n* = 6); EI (*n* = 5); favorable social behaviors and positive communication strategies (*n* = 5).

### Negative correlations to SI

The most frequently identified attributes significantly negatively correlated to SI were burnout and perceived stress (*n* = 5), as well as depression and anxiety (*n* = 5).

The full extent of attributes correlated to SI is reported in Table 3, along with the direction of said correlation (positive or negative).

**Table 3.** SI correlated attributes by major domains.

Human life domain	SI correlated attributes
Personal domain	resilience (+); happiness (+); emotional dysregulation (-); self-management (+); self-efficacy (+); personality traits; depression and anxiety (-); death anxiety (+); meaning in life (+); satisfaction in life (+); general, mental and spiritual health (+)
Social domain	forgiveness (+); empathy (+); favorable social behavior (+); communication skills (+); aggression (-), ethical thinking (+)
Work domain	work performance (+); academic/cognitive performance (+); job satisfaction (+); burnout and perceived stress (-); motivation (+); psychological ownership (+); commitment (+); professional self-concept (+); quality of work life (+)

(+) positive correlation; (-) negative correlation.

Globally addressing the main question of what is known about the influence of SI on human behavior and mental health, the gathered data suggests that SI is beneficial for mental health and for protecting against mental health risk factors, as well as being behaviorally well adaptive.

## Discussion

Since the first mentions about SI, in 1997 by Zohar [16], and throughout the concept’s journey into the Multiple Intelligences debate in the early 2000’s, the vast majority of the scholarly work on developing the concept and proving its criteria compliance with criteria as a separate type of intelligence has been conducted in North America [2,17–21].

After this initial theoretical approach and once the concept gained maturity to start being tested in its daily life impact, there was an interesting geographic shift of attention with most studies featuring quantitative research on SI being published from Asia and the Middle East.

The historical attention Eastern cultures dedicate to holistic practices, mindfulness and intuition, as along with the common fusion of religious practices and beliefs within cultural and political settings, may explain the readiness to investigate the SI concept in action [22].

Although the initial interest on SI applicability was scarce, as more evidence was gathered, interest rose exponentially, particularly after the recent pandemic generated deep existential concerns and heightened awareness of the importance of spiritual and mental health worldwide.

Despite the majority of the studies being from Eastern culture settings, the most frequently used instrument to evaluate SI was the originally Canadian SISRI-24 [2]. Aside from being easy to apply, this tool segregates from any religious connections, allowing it to be trustworthy in any cultural and/or religious setting and also accurate in evaluating SI in big culturally diverse samples.

It is well established in Gardner’s Multiple Intelligence Theory that one of the eight criteria for any type of intelligence should be its developmental history along the human lifespan, thus it is expected to grow from previous experiences and life challenges. Three different studies by Yang et al. [23–25], which included 953 nurses from Taiwan and China, helped explore SI demographics and yielded important conclusions. These studies not only confirmed its increase with age (as previously reported by King and DeCicco when validating the SISRI-24 scale [2]), but also revealed relevant associations with other demographic factors, such as religious affiliation and marital status.

Having some sort of religious belief or affiliation, even if not actively participating in religious rites and ceremonies, is also related to higher SI. Knowing that SI integrates dimensions of critical existential thinking and transcendental awareness, it is easily understandable that any sort of religious engagement may act as a trigger for such personal awareness, even if not essential for its development [19,23].

Higher SI was also related to married status and shared living situations and this may either come from a place of cause as of consequence, or both. When we further analyze the personal and social attributes related to SI, it is clear the implications and benefits for a shared existence scenario. For instance, higher SI relates to resilience, happiness, compassion, satisfaction in life, forgiveness, empathy, communication skills, commitment, less emotional dysregulation and less aggression. All the previous attributes can either be facilitators of a couple’s relationship and/or developed and enhanced through the marital partnership, increasing SI for married people [24,26].

The overall documented implications of SI increase the evidence supporting the necessity for a long-overdue revision of the World Health Organization (WHO) definition of health to adequately reflect the impact of this domain. If recurring arguments on the ambiguity of ‘spiritual’ and its relatability to ‘religion’ may be valid points against its inclusion on a revised definition of health, perhaps a different perspective is needed [27].

From a SI perspective, one can argue that everyone should be allowed and encouraged to freely and creatively explore existential matters, become aware of their spirituality and engage in it as they see fit. Furthermore, individuals should be encouraged to find meaning in both simple daily experiences and major life events, and to adopt a conscious metaview of life. These practices foster the self-awareness necessary for the development of SI, which has a significant impact on health and healthcare.

In fact, intervention studies aiming to improve SI in adolescents and university students have consistently reported both its success in increasing SI scores [28,29] and in reducing stress, anxiety and depression [29,30], proving the effectiveness and benefit of these interventions from early ages towards better mental health and healthier coping strategies.

On a personal level, SI has consistently been associated with resilience. The ability to find meaning in daily simplicity and to consider everyday events in the context of a bigger picture makes individuals more likely to accept adversity as a challenge and seek constructive and comforting problem-solving strategies. Different authors studied the relationship between SI and resilience with consistent significant positive correlations [31–33] and report prediction models with SI explaining variances from 10% [32] to 18% [34] on resiliency scores.

This capacity to find meaning in life in a broad sense but also in the smallest tasks, brings the also described higher sense of happiness (described SI-related variances of 19,8% [35] up to 67% [36]), higher life satisfaction [37] and fewer depression symptoms (SI accounting for 55% negative variance, according to Parattukudi et al. [38]).

The deeper self-knowledge and self-awareness cultivated through full attention to body, mind and soul/spiritual-self, enhance an individual’s self-management, self-efficacy and emotional self-regulation skills. These attributes not only contribute to stronger mental and spiritual health (as evidenced by reduced depression, anxiety, and death anxiety), but also foster a greater respect for the sacredness of the physical body and better health behaviors. Such behaviors have been shown to lead to better overall health outcomes, as demonstrated in the management of cardiovascular disease and diabetes management [39–41].

As SI grows from the singularity of one’s individual spirituality, it then projects widely in the ‘give and take’ of social interactions, becoming a practical aspect of one’s cognition. These social contexts are where the personal benefits of self-awareness and self-compassion transform into a metaview of life and relationships.

When SI is high, forgiveness and empathy run easily [42,43] A mind free from pre-conceptions and judgment, with sharp ethical thinking promoted by a multi-angled and multi-level vision of the problems, is easier to communicate with and has the plasticity to adjust to the interpersonal needs in different social settings [44,45]. These favorable social behaviors that come from the understanding and acceptance of a world full of personal truths and doubts, lessens the social anxiety (as shown by Mosavinezhad et al., SI determines 42,9% of social anxiety variation [11]) and aggressive behaviors (according to Ballochi et al., SI negative impact over aggression accounts for 29,5% of its variation [46]).

In the specific domain of the workplace, it is of utmost importance for the quality of work life that individuals can derive meaning from their labor and recognize the broader impact of their work. Singla et al. demonstrated a 49.5% variance in quality of work life related to SI among college teachers [47]. The spiritual capital one is able to draw from work enhances performance, with SI documented to impact performance ranging from 31.5% to 76.7% [9,48,49]. Additionally, organizational citizenship behaviors [50] and organizational commitment have reported a direct impact from SI of 10.8% according to Handayani et al. [48], and an indirect impact on employees through managers’ SI of 18% according to Dargahi and Veysi [51]. A recent scoping review on models of SI intervention programs suggests that 7–8 group sessions of 90 minutes, focusing mainly on transcendental awareness, critical existential thinking and conscious state expansion, through guided open discussions on ethical, existential and spiritual big issues and meditation or mindfulness exercises can benefit self-awareness, self-management and self-consciousness and also increase meaning in life and sense of holiness, promoting better relationships [52].

A particular subset of employees that would greatly benefit from such SI intervention programs being implemented in their pre-graduate curricula are the healthcare professionals. In a time of great advances in artificial intelligence, people crave for meaningful connections to each other, specially at times of vulnerability and disease as in grave illness or palliative care settings when existential matters are daily present for both patients and healthcare professionals. In fact, a recent systematic review on the benefits of SI training for nurses [53], consolidates evidence of significant increases in communication skills, spiritual care competence and job satisfaction, allied with reduced stress.

Nurses with higher SI also demonstrate a stronger professional self-concept, with 13.3% of the variance attributed to SI according to Hojat and Badiyepeymaiejahromi [54]. They also exhibit greater psychological ownership over their work (12,0% SI-related variance determined by Kaur et al. [12]), as they perceive their role within the universal flow of things.

This positive attitude around the workplace as well as the personal and social skills already explored that relate to higher SI, are consistently reported to prevent burnout and reduce perceived professional stress [12].

Besides personal advantages for healthcare workers, SI was also positively related to better caring behavior [12], spiritual care competency [26,55], ethical decision-making [44] and empathy [43] from nurses.

The above-mentioned scoping review on models of SI interventions [52] was also effective in compiling relevant data on the benefits of SI training for healthcare professionals. These interventions not only succeed in increasing SI levels but also result in post-intervention reductions in perceived stress, higher job satisfaction, improved spiritual care competence, and enhanced communication skills.

Healthcare professionals are very exposed to profound suffering, adversity, and challenges, encompassing not only physical and mental needs but also ethical and spiritual dilemmas. Thus, patients and their families need compassionate care, tailored to their individual and unique needs. Given the compelling arguments in favor of the positive implications of SI, it seems only fitting that this relevant topic finds its way into all fields of healthcare education curricula [56], benefiting both healthcare workers and patients.

## Strengths and limitations

The major strengths of this ScR come from its broad search strategy and systematic methodology, which allowed for a thorough mapping of the existing evidence on SI. The quality of the reported evidence was also guaranteed through double independent analysis and methodology check of all screened manuscripts by two independent reviewers. Last, but not least, we proceeded to a careful integration and interpretation of the results in the light of the current knowledge.

Although our search included different languages (English, French, Spanish, and Portuguese), the subsequent identification of significant contributions to the study of SI from Oriental and Arabic countries may have limitations, as it may have missed publications in these languages. Since no grey literature was included for analysis, there is a possibility that early evidence on SI or evidence from areas of knowledge that do not typically undergo peer review may have been overlooked.

## Conclusion

Integrating basic SI stimulation interventions into global children’s educational programs, from early years to the higher education (specially in healthcare professions), may be the simplest, most efficient and cost-effective approach to ensure a stronger mental health foundation for our future generations. Additionally, it can promote healthy coping mechanisms, health-related behaviors, and foster the development of more compassionate and tolerant adults.

From the above-exposed evidence, it is clear that SI training is effective in increasing SI levels and brings along major benefits towards general health, mental and spiritual health.

Anticipated main challenges toward implementing SI stimulation interventions include the risk of them being overshadowed by religious perspectives. Hence, we propose that general recommendations for SI training programs, encompassing both content and application, be developed through culturally diverse expert consensus. These interventions should always be supervised by a selected group of counselors who can ensure that the training protocols are being adequately applied. Pre-test/post-test evaluations should also be undertaken to provide further evidence of the adequacy of the protocol and to facilitate additional research and exploration of the impact of SI interventions.
